# Correlation of troponin I and CK-MB with COVID-19 severity in seriously ill patients: A retrospective analysis from medical records

**DOI:** 10.6026/973206300214585

**Published:** 2025-12-15

**Authors:** Jyoti Shukla, Ritesh Yadav, Divyansh Gupta, Pankaj Sharma, Nishant Singh Verma

**Affiliations:** 1 Department of Biochemistry, Shrimant Rajmata Vijayaraje Scindia Medical College Shivpuri, Madhya Pradesh, India; 2 Department of General Medicine, Shrimant Rajmata Vijayaraje Scindia Medical College Shivpuri, Madhya Pradesh, India; 3 Department of Orthopaedics, Shrimant Rajmata Vijayaraje Scindia Medical College, Shivpuri, Madhya Pradesh, India

**Keywords:** COVID-19, SARS-CoV-2, Myocardial Injury, Tr, CK-MB, Prognosis, Severity

## Abstract

COVID-19 is frequently associated with cardiovascular complications, with cardiac troponin I (cTnI) and CK-MB serving as markers of myocardial injury. Therefore, it is of interest to analyse of medical records from 300 PCR-confirmed COVID-19 patients admitted to High Dependency Unit (HDU) and Intensive Care Unit (ICU) during the second wave (2020-2021), biomarker levels at admission were correlated with disease severity. All patients included were seriously ill but survived. Severe cases had significantly higher cTnI and CK-MB levels (p < 0.001). These biomarkers provide important early prognostic information For the purpose of risk assessment and therapeutic treatment of COVID-19.

## Background:

The coronavirus disease 2019 (COVID-19) pandemic, caused by severe acute respiratory syndrome coronavirus 2 (SARS-CoV-2), has posed an unprecedented global health challenge. Although COVID-19 primarily manifests as a respiratory illness, research indicates it is a multi-systemic disease with significant extrapulmonary manifestations [1, 2]. Among these complications, cardiovascular issues are prominent and contribute substantially to morbidity [3, 4]. The presence of acute myocardial damage is consistently linked to worse prognosis and is observed in a significant percentage of hospitalized patients, particularly those with severe illness [5]. According to the Fourth Universal Definition of Myocardial Infarction, cardiac troponin values must demonstrate an increase or decrease with at least one value above the 99th percentile upper reference limit (URL) to be considered clinically significant [6]. The pathophysiological mechanisms of COVID-19-induced myocardial injury are multifaceted. One proposed pathway involves direct viral infection of cardiomyocytes via ACE2 receptors, which are abundantly expressed in cardiac tissue [7, 8]. Additionally, systemic inflammation and cytokine storms can induce myocyte apoptosis and endothelial dysfunction [9, 10]. Other contributing factors include hypoxia-induced cellular damage from severe respiratory failure, a prothrombotic state leading to microvascular thrombosis and stress-induced cardiomyopathy [11, 12]. Cardiac troponin I (cTnI) and creatine kinase-myocardial band (CK-MB) are reliable markers for identifying myocardial necrosis. cTnI is highly sensitive and specific for cardiac muscle damage, making it the cornerstone for diagnosing acute myocardial injury and a key prognostic indicator [13]. CK-MB, while less specific, remains a valuable adjunct marker [14]. Early studies from Wuhan, China, highlighted the concerning association between elevated cardiac troponins and adverse outcomes in COVID-19 patients [15]. Subsequent research from the US and Europe confirmed these findings, showing that myocardial injury is a strong predictor of unfavorable outcomes [16, 17- 18]. Large-scale meta-analyses have consolidated this evidence, confirming the prognostic value of troponin across diverse populations and clinical settings [19, 20]. However, a research gap remains in understanding the comparative prognostic utility of cTnI versus CK-MB at initial presentation, especially in seriously ill patients who survive, accounting for regional variations in patient demographics and comorbidity burden [21]. Early and accurate risk stratification is crucial for optimizing clinical management, including decisions regarding level of care and initiation of potential cardioprotective therapies [22, 23]. Therefore, it is of interest to determine the correlation between cTnI and CK-MB levels at admission and disease severity in seriously ill COVID-19 patients admitted to HDU and ICU who survived, using data collected from medical records during the second wave of the pandemic (2020-2021).

## Materials and Methods:

## Study design and population:

We conducted a retrospective analysis of medical records from adult patients hospitalized with confirmed SARS-CoV-2 infection via nasopharyngeal swab and reverse transcriptase-polymerase chain reaction (RT-PCR) during the second wave of the pandemic (April 2020 to March 2021). The study included 300 patients who fulfilled the inclusion criteria. To be included, patients needed to meet three conditions: (1) age of at least 18 years; (2) positive SARS-CoV-2 RT-PCR test result; and (3) admission to HDU or ICU; (4) survival to hospital discharge. Exclusion criteria were: (1) patients with acute coronary syndrome within 30 days before admission; (2) patients with end-stage renal disease (eGFR <15 mL/min/1.73 m^2^) or on chronic dialysis; (3) patients with severe traumatic injury on admission; (4) patients with incomplete records for primary outcomes or important laboratory data; and (5) patients who died during hospitalization.

## Data collection and definitions:

Data were extracted from the institutional Medical Records Department database. Collected data included patients' demographic information (age, sex), medical history (hypertension, diabetes mellitus, coronary artery disease, chronic obstructive pulmonary disease, obesity) and clinical outcomes (disease severity, length of stay in HDU/ICU, need for mechanical ventilation). Disease severity was categorized using the World Health Organization (WHO) clinical progression scale. Patients were dichotomized into: Moderate (requiring supplemental oxygen but not high-flow oxygen or non-invasive ventilation) and Severe (requiring high-flow oxygen, non-invasive ventilation, or mechanical ventilation).

## Laboratory analysis:

Serum cTnI levels were assessed using a high-sensitivity assay, with the 99th percentile upper reference limit (URL) of 0.04 ng /mL considered the threshold for myocardial damage. CK-MB levels were quantified using standard methods, with a normal reference range of 0-24 U/L.

## Statistical analysis:

All statistical analyses were performed using IBM Corp.'s SPSS Statistics for Windows, Version 26.0. Continuous variables were presented as mean ± standard deviation (SD) and compared using the independent Student's t-test or Mann-Whitney U test. Categorical variables were reported as frequency (%) and compared using the Chi-square test or Fisher's exact test. A two-sided p-value < 0.05 was considered statistically significant. Multivariate logistic regression analysis was conducted to identify independent determinants of disease severity after adjusting for age, sex and significant comorbidities.

## Results:

A total of 300 COVID-19-positive patients admitted to HDU or ICU who survived were included in the analysis. The mean age was 58.7 ± 14.3 years and 168 (56.0%) were male. Upon admission, 125 (41.7%) were classified as having moderate disease, while 175 (58.3%) were classified as having severe disease. Patients in the severe group were significantly older (64.2 ± 12.1 years vs. 51.3 ± 14.7 years, p < 0.001) and had a higher prevalence of comorbidities, including hypertension (62.3% vs. 45.6%, p = 0.003) and diabetes mellitus (38.9% vs. 24.8%, p = 0.011). Baseline characteristics are detailed in Table 1 (see PDF). Patients with severe COVID-19 had significantly deranged laboratory parameters. As shown in Table 2 (see PDF), the mean admission cTnI level in the severe group was over three times higher than in the moderate group (0.24 ± 0.14 ng/mL vs. 0.07 ± 0.05 ng/mL, p < 0.001). Mean CK-MB levels were also significantly elevated in the severe group (28.7 ± 12.5 U/L vs. 14.2 ± 7.3 U/L, p < 0.001). A higher proportion of patients with severe disease had cTnI levels above the URL (82.3% vs. 32.0%, p < 0.001). In a multivariate logistic regression model adjusted for age, hypertension and diabetes, an admission cTnI level >0.04 ng/mL remained an independent predictor of severe disease (Odds Ratio: 3.8, 95% CI: 2.1-6.9, p < 0.001).

## Discussion:

In this retrospective analysis of medical records from seriously ill COVID-19 patients admitted to HDU and ICU who survived, we demonstrated a strong and clinically significant correlation between elevated levels of cTnI and CK-MB at admission and disease severity. Our main finding is that these biomarkers show a robust association between myocardial injuries at initial evaluation and worsening disease severity, need for mechanical ventilation and longer duration of stay in HDU/ICU. Our results align with existing literature [15, 16, 17, 18-19]. Our finding that elevated cTnI on admission is an independent predictor of severe disease (OR: 3.8) is quantitatively similar to results reported by other large cohorts. The pathophysiology linking myocardial injury to COVID-19 severity is complex and multifactorial. High concentrations of interleukin-6 and other inflammatory mediators, known as the "cytokine storm," may cause direct damage to cardiomyocytes [10]. Both direct viral infiltration and inflammation-mediated heart injury have been demonstrated in histopathological investigations [24, 25]. Furthermore, the widespread endotheliitis and procoagulant state associated with severe COVID-19 can precipitate microthrombi formation in the cardiac microvasculature, exacerbating ischemic injury [11, 26]. Our findings have several important clinical implications. Testing for cTnI and CK-MB is widely accessible and can be performed rapidly. Our data support the integration of these biomarkers into initial risk assessment protocols for COVID-19 patients, a recommendation echoed by several cardiology societies and expert consensus statements [22, 27]. Early identification of myocardial injury may allow for the timely implementation of cardioprotective strategies and more vigilant hemodynamic monitoring, potentially improving outcomes in seriously ill patients [28]. Several limitations should be considered. First, the retrospective, single-center design may limit generalizability. Second, we did not systematically distinguish between Type 1 and Type 2 myocardial injury, a common limitation in COVID-19 studies [29, 30]. Third, although we adjusted for significant comorbidities, residual confounding from unmeasured covariates remains possible. Finally, our study focused only on patients who survived; therefore, we cannot extrapolate our findings to predict mortality.

## Conclusion:

Elevated levels of cardiac troponin I and CK-MB at admission are significantly associated with increased disease severity among seriously ill COVID-19 patients admitted to HDU and ICU who survived. Thus, myocardial injury is a serious concern in severe SARS-CoV-2 infection. Regular testing of these biomarkers is a simple yet effective method for early risk stratification, helping clinicians identify high-risk patients who may benefit from more intensive monitoring and treatment.
